# Increased middle cerebral artery velocity predicts malignant media infarction after endovascular stroke thrombectomy

**DOI:** 10.1177/17562864251374935

**Published:** 2025-10-02

**Authors:** Enayatullah Baki, Victoria Kehl, Marlene Topka, Felix Hess, Sebastian Lambrecht, Bernhard Hemmer, Silke Wunderlich, Johanna Haertl

**Affiliations:** Department of Neurology, School of Medicine and Health, Klinikum Rechts der Isar, Technical University of Munich, Ismaninger Str. 22, Munich 81675, Germany; Institute of AI and Informatics in Medicine, School of Medicine and Health, Klinikum Rechts der Isar, Technical University of Munich, Munich, Germany; Department of Neurology, School of Medicine and Health, Klinikum Rechts der Isar, Technical University of Munich, Munich, Germany; Department of Neurology, School of Medicine and Health, Klinikum Rechts der Isar, Technical University of Munich, Munich, Germany; Department of Neurology, School of Medicine and Health, Klinikum Rechts der Isar, Technical University of Munich, Munich, Germany; Department of Neurology, School of Medicine and Health, Klinikum Rechts der Isar, Technical University of Munich, Munich, Germany; Munich Cluster for Systems Neurology, Munich, Germany; Department of Neurology, School of Medicine and Health, Klinikum Rechts der Isar, Technical University of Munich, Munich, Germany; Department of Neurology, School of Medicine and Health, Klinikum Rechts der Isar, Technical University of Munich, Munich, Germany

**Keywords:** endovascular thrombectomy, ischemic stroke, transcranial Duplex sonography

## Abstract

**Background:**

Increased peak systolic velocity (PSV) in transcranial Doppler or Duplex sonography (TCD) of the middle cerebral artery (MCA) after endovascular thrombectomy (EVT) for large vessel occlusion in acute ischemic anterior circulation stroke has been associated with poor functional outcome and increased risk of symptomatic intracranial hemorrhage (ICH).

**Objective:** We evaluated whether increased MCA-PSV is associated with the development of malignant media infarction after EVT.

**Methods:**

We retrospectively identified all patients who underwent EVT for acute anterior circulation ischemic stroke at our stroke center from January 2021 to July 2024. Increased MCA-PSV on TCD was defined as >30% mean PSV in the treated MCA compared with the contralateral MCA. The development of malignant media infarction was evaluated according to predefined clinical and neuroimaging criteria. Multivariable regression models were used to identify associations between MCA-PSV and the development of malignant media infarction.

**Results:**

Out of a total cohort of 377 patients, 49 (13.0%) developed malignant media infarction. In multivariable analysis, MCA-PSV increase was significantly associated with malignant media infarction (odds ratio (OR), 53.3 (95% confidence interval (CI): 18.74, 151.54); *p* < 0.001). Furthermore, the development of malignant media infarction was also associated with secondary ICH (OR, 6.4 (95% CI: 2.16, 19.03); *p* < 0.001) and higher baseline National Institutes of Health Stroke Scale (OR, 1.25 (95% CI: 1.14, 138); *p* < 0.001).

**Conclusion:**

Increased MCA-PSV can act as a predictive marker for the development of malignant media infarction. TCD may serve as a valuable bedside tool in individual risk assessment in early postinterventional surveillance.

## Background

Malignant middle cerebral artery (MCA) infarction after endovascular thrombectomy (EVT) for acute ischemic stroke is a severe complication associated with high mortality.^1^ Early detection of malignant infarction is highly relevant in identifying patients who may benefit from surgical treatment by decompressive hemicraniectomy.^2–6^ Various risk factors for developing malignant MCA infarction have been previously described. These include lower patient age, large magnetic resonance imaging (MRI) diffusion-weighted imaging core volume, lower Alberta stroke program early computed tomography (CT) score, proximal artery occlusion including MCA and internal carotid artery (ICA) occlusion, lower collateral score, longer times from onset to groin puncture, unsuccessful reperfusion defined as an expanded treatment in cerebral infarction (eTICI) score of 0–2a, elevated S100B as a serum biomarker, and a severe deficit assessed by the National Institutes of Health Stroke Scale (NIHSS) at initial presentation.^7–12^

Increased peak systolic velocity (PSV) of the ipsilateral MCA, measured by transcranial Doppler or Duplex sonography (TCD), is associated with poor neurological outcome and intracranial hemorrhage (ICH) after EVT.^13–16^ Additionally, MCA-PSV increase caused by cerebral vessel wall injury, de novo or persisting stenosis after EVT, and vasospasm have been reported.^17,18^ Even though associations between MCA-PSV and poor neurological outcomes as well as the development of vasogenic edema on MRI have been described,^13^ the association between MCA-PSV and the development of malignant media infarction remains unexplored. Hence, we aimed to assess whether TCD of the ipsilateral MCA after EVT may serve as a bedside diagnostic tool for risk stratification in patients developing malignant cerebral infarction.

## Methods

### Data source, patient selection, and cohort development

In a retrospective approach, we identified all patients admitted to our certified stroke care center who received EVT for acute ischemic stroke caused by ICA and/or MCA occlusion between January 2021 and July 2024 from the local stroke quality assurance registry (Bavarian Working Group for Quality Assessment, BAQ) with available TCD recordings performed within 72 h after thrombectomy. We excluded all patients with ipsi- or contralateral MCA stenosis, with or without MCA stenting, diagnosed with CT angiography, MR angiography or digital subtraction angiography, and missing or insufficient temporal bone window.

### Demographic and clinical characteristics

Patient demographic and clinical characteristics included age, sex, NIHSS on admission, side of the occluded vessel, intravenous thrombolysis with tissue plasminogen activator (alteplase), failed revascularization (TICI 0–2a), carotid artery stenting, and the number of passes during mechanical thrombectomy. As postinterventional complication, secondary intracerebral hemorrhage (ICH), defined as a distinct, nontraumatic bleeding into the parenchyma^19^ was evaluated for each patient. The PSV of the ipsilateral (recanalized) MCA was retrospectively analyzed both as a continuous variable (cm/s) and as a dichotomous variable (PSV increase) by two independent experienced neurologists (E.B., J.H.). Increased MCA-PSV on TCD was defined as >30% mean PSV in the treated MCA compared with the contralateral MCA.^13^

### Endpoint

The endpoint was the development of malignant media infarction based on the following clinical and imaging criteria: Clinical signs of large MCA territory infarction with a NIHSS score >18 points and a level of consciousness of ⩾1 on item 1a of the NIHSS, and large space-occupying MCA infarction on follow-up MRI or CT of at least two-thirds of the MCA territory with compression of the ventricles or midline shift, and no other apparent cause for neurological deterioration.^7^

### Statistical analysis

All statistical analyses were performed using IBM SPSS Statistics for Windows (version 29.0.2.0, IBM Corp., Armonk, NY, USA). Descriptive statistics were presented as frequencies with percentages for categorical variables, as means with standard deviations for approximately normally distributed continuous variables, and as median with range for ordinal or not normally distributed variables as appropriate. Descriptive statistics were provided overall and by subgroups of patients with and without malignant media infarction. To identify potential predictors of malignant media infarction, we used univariate and multivariable logistic regression models. We presented the results using odds ratio (OR), its 95% confidence interval (CI), and the *p*-value of the Wald statistic. All statistical tests were performed two-sided at the global 5% significance level. The Bonferroni–Holm correction was used to adjust the *p*-values for multiple testing during the univariate analysis.

## Results

### Patient cohort

**Figure 1.**
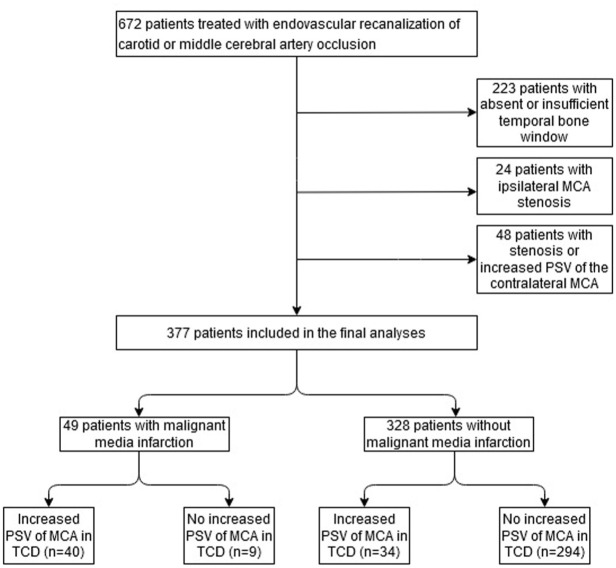
Study flow chart.

**Figure 2.**
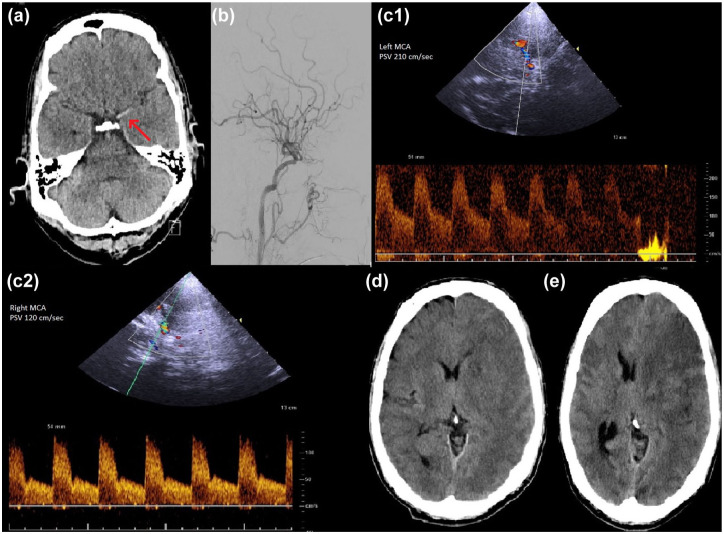
Exemplary case of a patient with right hemiparesis and aphasia (NIHSS score 19 points). (a) Initial CT scan showing hyperdense media sign (red arrow), proximal occlusion of the left MCA. (b) Digital subtraction angiography after successful mechanical recanalization of the left MCA (TICI 2c). (c1) Transcranial Duplex sonography of the left MCA with increased PSV (210 cm/s) 5 h after recanalization compared to (c2) the right MCA. (d, e) CT scans showing malignant swelling of MCA infarction with midline shift 6 and 16 h after successful recanalization. MCA, middle cerebral artery; NIHSS, National Institutes of Health Stroke Scale; PSV, peak systolic velocity; TICI, thrombolysis in cerebral infarction.

In total, 672 patients underwent EVT for acute ischemic stroke caused by ICA and/or MCA occlusion from January 2021 to July 2024 at our stroke center. According to the above-defined exclusion criteria, 295 patients were excluded from statistical analysis, resulting in a cohort of 377 patients, of which 49 (13.0%) developed malignant media infarction. The study flow chart is depicted in Figure 1. Patients included in the study had a mean age of 72.2 years. The median NIHSS at admission was 13 points, ranging from 0 to 33 points. 288 of 377 patients (76.4%) were treated for isolated MCA occlusion, 52 patients (13.8%) for isolated ICA occlusion, and 37 patients (9.8%) for combined ICA/MCA occlusion. A total of 163 patients (43.2%) received bridging intravenous thrombolysis. In total, 74 patients (19.6%) showed postinterventional MCA-PSV elevation. All patient characteristics are displayed in Table 1. Figure 2 shows an exemplary case of a patient with increased MCA-PSV after successful mechanical recanalization of an MCA occlusion with malignant media infarction on CT scan after sonography.

**Table 1.** Comparison of demographic, clinical, and sonographic characteristics between patients after endovascular MCA/ICA thrombectomy with and without the development of malignant media infarction.

	Total cohort (*n* = 377)	Malignant media infarction (*n* = 49)	No malignant media infarction (*n* = 328)	OR	95% CI for OR	*p*-Value^a^	Adjusted *p*-value^b^
Demographic characteristics							
Age (years), mean (±SD)	72.2 (±14.7)	74.0 (±13.2)	72.0 (±14.9)	1.010	(0.988, 1.032)	0.336	>0.999
Female, *n* (%)	150 (39.8%)	21 (42.9%)	129 (39.3%)	1.157	(0.630, 2.124)	0.638	>0.999
Acute stroke severity index							
NIHSS on admission, median (range)	13 (0–33)	19 (8–33)	12 (0–25)	1.248	(1.164, 1.338)	<0.001	<0.001
Occluded vessel, *n* (%)							
MCA	288 (76.4%)	31 (63.3%)	257 (78.4%)	0.476	(0.252, 0.900)	0.022	0.308
ICA	52 (13.8%)	14 (28.6%)	38 (11.6%)	3.053	(1.507, 6.184)	0.002	0.028
ICA + MCA	37 (9.8%)	4 (8.2%)	33 (10.1%)	0.795	(0.269, 2.349)	0.678	>0.999
Occlusion on the right side	167 (44.3%)	23 (46.9%)	144 (43.9%)	1.130	(0.619, 2.064)	0.690	>0.999
Therapy, *n* (%)							
Intravenous tPA	163 (43.2%)	20 (40.8%)	143 (43.6%)	0.892	(0.485, 1.642)	0.892	>0.999
Failed revascularization (TICI 0–2a)	27 (7.2%)	1 (2.0%)	26 (7.9%)	0.242	(0.032, 1.825)	0.169	>0.999
Carotid artery stenting	57 (15.1%)	5 (10.2%)	52 (15.9%)	0.603	(0.228, 1.593)	0.308	>0.999
Number of passes, mean (±SD)	2.1 (±1.4)	2.6 (±1.5)	2.0 (±1.4)	1.305	(1.080, 1.578)	0.006	0.084
Complication, *n* (%)							
ICH	71 (18.8%)	18 (36.7%)	53 (16.2%)	3.013	(1.571, 5.777)	<0.001	0.013
Postintervention TCD							
Ipsilateral (recanalized) MCA: mean PSV (cm/s), mean (±SD)	97.9 (±41.6)	143.3 (±45.3)	91.1 (±36.5)	1.027	(1.019, 1.035)	<0.001	<0.001
Increased MCA-PSV, *n* (%)	74 (19.6%)	40 (81.6%)	34 (10.4%)	38.431	(17.173, 86.007)	<0.001	<0.001

a *p*-Value of the Wald statistic from the corresponding univariate logistic regression model with dependent variable malignant media infarct (yes/no).

b *p*-Value of the Wald statistic from the corresponding univariate logistic regression model with dependent variable malignant media infarction (yes/no), adjusted for multiple testing.

### Univariate and multivariable regression models with the endpoint malignant media infarction

In univariate analysis, NIHSS at admission (*p* < 0.001), isolated ICA occlusion (*p* = 0.028), and postinterventional ICH (*p* = 0.013), were associated with the development of malignant media infarction.

Mean MCA-PSV in patients with malignant media infarction was measured at 143.3 ± 45.3 cm/s, while mean MCA-PSV in patients without malignant media infarction was at 91.1 ± 36.5 cm/s (*p* < 0.001). Of all 49 patients who developed malignant infarction, 40 (81.6%) showed an MCA-PSV elevation >30% in relation to the contralateral MCA compared to 34 of 328 (10.4%) patients without malignant infarction (*p* < 0.001).

The multivariable analysis was first performed, including all variables that were significant in the univariate models at the 5% level after adjustment for multiple testing. The variables “isolated ICA occlusion” and “mean PSV” were not significant in the multivariable setting and therefore excluded from the final multivariable model. Notably, the Spearman’s correlation coefficient between mean PSV and increased PSV was 0.646 with *p* < 0.001. Both variables bring similar information into the model. Increased PSV is the stronger predictor between them. Once the stronger variable is in the model, the continuous variable “mean PSV” does not bring any additional information into the model and is, therefore, no longer significant. The variable “isolated ICA occlusion” is simply weaker than the rest and cannot add any more information to the model.

In the final multivariable analysis, NIHSS at admission, secondary ICH, and increased MCA-PSV remained significantly associated with the development of malignant infarction with a *p*-value <0.001 for each variable, respectively. The results of the multivariable logistic regression analysis are presented in Table 2.

**Table 2.** Multivariable logistic regression predicting malignant media infarction.

Variables	OR	95% CI for OR	*p*-Value^a^
NIHSS on admission	1.250	(1.135, 1.377)	<0.001
ICH	6.411	(2.160, 19.027)	< 0.001
Increased MCA-PSV	53.289	(18.739, 151.543)	< 0.001

a *p*-Value of the Wald statistic from the corresponding multivariable logistic regression model with dependent variable malignant media infarction (yes/no).

CI, confidence interval; ICH, intracerebral hemorrhage; MCA, middle cerebral artery; NIHSS, National Institutes of Health Stroke Scale; OR, odds ratio; PSV, peak systolic velocity.

## Discussion

We provide the first study identifying an association between increased PSV of the ipsilateral MCA on TCD after EVT and the development of malignant media infarction.

Increased MCA-PSV after EVT has previously been associated with a worse neurological outcome at 90 days and the development of symptomatic ICH.^13–16^ Furthermore, it has been associated with vasogenic edema on MRI.^13^ Regarding infarct size, contradictory results have been shown: While in a prospective patient cohort, Kneihsl et al.^13,15^ showed a significant association of MCA-PSV and infarct size, the same study group did not detect a significant association of abnormal TCD findings and an infarct size of >2/3 of MCA territory. In our patient cohort, elevated MCA-PSV was significantly associated with development of malignant media infarction. Taking our study findings in the context of the existing literature, we assume that the described association between sonographic alterations and poor outcomes might be, in fact, at least partially ascribed to the development of malignant media infarction but has not been yet sufficiently explored.

Different pathomechanisms underlying the hemodynamic changes of recanalized vessels and elevated MCA-PSV have been discussed including altered autoregulation, cerebral vessel wall injury, de novo or persisting stenosis, and vasospasm.^14,16–18^ Impaired auto regulation, as assessed by cerebral perfusion pressure-oxygen reactivity within 24 and 72 h as well as by TCD, has been described as a risk factor for a malignant course with massive edema formation.^20–22^ Moreover, we consider local space-occupying effects mediated by malignant swelling of the infarction as causative for MCA-PSV increase due to compression of the intracranial arteries.

The overlap of malignant media infarction and ICH in our patient cohort is likely based on the higher risk of ICH in large infarctions. Previous studies characterized a strong association between hemorrhagic transformation of cerebral infarction and infarct size.^23–27^ The disruption of the blood–brain barrier after large infarction and reperfusion injury causes leakage of peripheral blood cells, especially after reperfusion therapy. Cerebral infarction also results in damage to capillary cells, which cause an increase in vascular permeability and extravasation of blood in the brain parenchyma.^25,28^

Limitations mainly refer to our retrospective study design. Our analyses depend on clinical data from medical records that were not primarily collected for research purposes. Larger prospective studies are required on this issue with clear protocol-defined time points for clinical, sonographic, and radiological assessments. Nonetheless, our work is the first study about postintervention sonography with malignant infarction as the endpoint, highlighting the importance of sonographic neuromonitoring as a valuable and feasible diagnostic after stroke thrombectomy.

In conclusion, elevated MCA-PSV is associated with malignant media infarction, and TCD may serve as a bedside tool for early recognition of patients at risk for this severe postinterventional complication.

## Declarations

**Ethics approval and consent to participate:** This study was performed in accordance with the principles of the Declaration of Helsinki. Ethical approval was granted by the Ethics Committee of the Technical University of Munich, ID: 2024-438-S-SB. Due to the retrospective study design and data anonymization, patient consent was not required.

**Consent for publication:** Not applicable.

**Availability of data and materials:** Data are available upon reasonable request.
